# Quantitative Dixon and intravoxel incoherent motion diffusion magnetic resonance imaging parameters in lumbar vertebrae for differentiating aplastic anemia and acute myeloid leukemia

**DOI:** 10.3389/fonc.2023.1277978

**Published:** 2023-12-04

**Authors:** Meidan Hou, Yanan Huang, Jinsong Yan, Guoguang Fan

**Affiliations:** ^1^ Department of Radiology, The First Hospital of China Medical University, Shenyang, China; ^2^ Department of Radiology, The Second Hospital of Dalian Medical University, Dalian, China; ^3^ Department of Hematology, Liaoning Medical Center for Hematopoietic Stem Cell Transplantation, Liaoning Key Laboratory of Hematopoietic Stem Cell Transplantation and Translational Medicine, The Second Hospital of Dalian Medical University, Dalian, China

**Keywords:** acute myeloid leukemia, aplastic anemia, intravoxel incoherent motion, quantitative Dixon, MRI

## Abstract

**Objective:**

We sought to evaluate the use of quantitative Dixon (Q-Dixon) and intravoxel incoherent motion diffusion imaging (IVIM) for the differential diagnosis of aplastic anemia (AA) and acute myeloid leukemia (AML).

**Methods:**

Between August 2021 and October 2023, we enrolled 68 diagnosed patients, including 36 patients with AA and 32 patients with AML, as well as 26 normal controls. All patients underwent 3-Tesla magnetic resonance imaging, which included IVIM and T2*-corrected Q-Dixon imaging at the L2–4 level. The iliac crest biopsy’s pathology was used as the diagnostic criterion. The interobserver measurement repeatability was evaluated using the intraclass correlation coefficient (ICC). One-way analysis of variance, Spearman analysis, and receiver operating characteristic curve analysis were used.

**Results:**

The fat fraction (FF) and perfusion fraction (f) values were statistically significantly different between the three groups (*p* < 0.001 and *p* = 0.007). The FF and f values in the AA group were higher than those in the AML group. The true apparent diffusion coefficient (D) value was substantially negatively correlated to the FF and R2* values (r = −0.601, *p* < 0.001; r = −0.336, *p* = 0.002). The f value was positively correlated with both FF and pseudo-apparent diffusion coefficient (D*) values (r = 0.376, *p* < 0.001; r = 0.263, *p* = 0.017) and negatively correlated with the D value (r = −0.320, *p* = 0.003). The FF and f values were negatively correlated with the degree of myelodysplasia (r = −0.597, *p* < 0.001; r = −0.454, *p* = 0.004), and the D value was positively correlated with the degree of myelodysplasia (r = 0.395, *p* = 0.001). For the differential diagnosis of AA and AML, the Q-Dixon model’s sensitivity (93.75%) and specificity (84%) confirmed that it outperformed the IVIM model.

**Conclusion:**

Q-Dixon parameters have the potential to be used as new biomarkers to differentiate AA from AML.

## 1 Introduction

Aplastic anemia (AA) is an acquired bone marrow failure characterized by pancytopenia and destruction of hematopoietic cells. AA has a low incidence, with two peaks during the lifespan in young and older people, respectively. The pathological features of AA are a decrease in hematopoietic tissue and the gradual replacement of red bone marrow by adipose tissue, which significantly modifies the composition of the bone marrow; when severe pancytopenia and secondary infections occur, the mortality is high (1, 2). AA can be divided into non-severe AA(NSAA)and severe AA(SAA) according to the hemogram and degree of bone marrow hyperplasia. When non-severe AA progresses to severe AA, it can manifest as severe anemia, infection, and bleeding in the brain and other vital organs, making it one of the major causes of death in AA cases (3). There is a lack of conservative and effective treatments for severe AA. Without the combination of immune-intensive therapy and allogeneic bone marrow transplantation, the mortality rate is very high (4).

Although AA is a benign disease, 10%–15% of patients experience malignant clonal disease evolution during the course of disease progression, especially those with acute myeloid leukemia (AML) and myelodysplastic syndrome (MDS). AA and low proliferative AML have similar peripheral blood manifestations. As mentioned, the pathological features of AA are a decrease in the hematopoietic tissue and gradual replacement of red bone marrow by adipose tissue (5–7). Separately, the pathogenic aspect of AML is the replacement of healthy hematopoietic tissue by tumor tissue, which causes the bone marrow’s fat content to drop and its internal vascular components to increase (8). The diagnosis of AA or AML is currently made based on bone marrow cell morphology, cellular immunotyping, and cytogenetic analysis. However, these techniques often take a long time, making it difficult to complete the differential diagnosis between AA and AML in a speedy manner, thus delaying the start of treatment. This, in turn, leads to an increase in mortality (9, 10). Bone marrow puncture or biopsy can only reveal the state of small local bone marrow, but cannot reflect the hematopoiesis of bone marrow in a large range, and there is sampling error (11). Due to the large number of protons in the fat and water of the blood and stromal tissues in the bone marrow, magnetic resonance imaging (MRI) can be an objective and non-invasive method to assess and characterize bone marrow, with great advantages in quantifying the bone marrow composition (12). Thus, combining bone marrow puncture and pathological biopsy would yield a more accurate and comprehensive systematic assessment of AA and AML (13, 14).

Bone marrow signal differences between AA and AML can be detected by conventional MRI, and the characteristics of T1-weighted, T2-weighted, and short tau inversion recovery signals are high signal and slightly high signal, low signal and low signal, and high signal and slightly high signal, respectively (13). However, conventional MRI cannot facilitate quantitative analysis of bone marrow, and there is also observer subjectivity. Several studies have used the relative fat content obtained by chemical shift imaging for diagnosis and efficacy assessment of AA and AML (13) and for post-treatment efficacy assessment of multiple myeloma (15). However, chemical shift imaging is highly dependent on magnetic field strength and homogeneity, and inhomogeneity of the static magnetic field B0 can easily lead to errors in quantitative analysis and calculations.

Quantitative Dixon (Q-Dixon) a six-echo–based water–lipid separation technique that allows accurate quantitative analysis of fat content in the bone marrow by correcting for the T2*-shortening effect caused by the bone trabeculae (16–18). It is widely used to differentiate between benign and malignant spinal lesions (19). The Q-Dixon technique has been used to measure the fat fraction (FF) of vertebral lesions, which can be used to distinguish benign and malignant lesions, given that the yellow bone marrow composition of benign and malignant vertebral lesions is different. Zeng et al. (20) observed that the fat content of AA was much greater than that of MDS using the IDEAL IQ sequence to identify AA from MDS. This may be because the hematopoietic cells in AA are replaced by adipose tissue and, when compared to normal bone marrow tissue, the mesenchymal stem cells of AA patients are more likely to stimulate adipocyte development (21), increasing the amount of fat in their bone marrow.

Intravoxel incoherent motion (IVIM) MRI can distinguish the diffusion and perfusion effects of simple water molecules in tissues through multiple b-values and help facilitate quantitative analysis (16, 22). Quantitative evaluation of diffusion and perfusion of yellow bone marrow and abnormal hematopoietic tissues in the bone marrow has been widely used to assess the therapeutic efficacy of AML. multiple myeloma and in the differential diagnosis of anemia and AML (6, 15, 22, 23).

Here, we evaluated the ability of the Q-Dixon and IVIM MRI parameters to distinguish between AA and AML.

## 2 Materials and methods

Our institutional review board approved this retrospective study, and the need for written informed consent was waived.

### 2.1 Study population

From August 2021 to October 2023, 68 patients with AA or AML diagnosed based on bone marrow aspirate and 26 health controls were recruited from Second Hospital of Dalian Medical University in Dalian, China. All patients included were older than 18 years, and who had the ability to lie still for more than 20 min and to cooperate with MRI. Patients with contraindications to MRI (e.g., claustrophobia, cardiac pacemaker, metal implants in the lumbar region, etc.) were not enrolled. Separately, patients treated with hematopoietic stem cell transplantation, patients with other malignant tumors or vertebral lesions, and patients with poor image quality were excluded.

### 2.2 Degree of bone marrow hyperplasia classification

Histological analysis was performed by a laboratory scientist with 10 years of experience in hematological disorders. Iliac/thoracic bone marrow aspiration was used for cytological analysis. Bone marrow cytosol was examined through a smear and staining process, starting with a low-magnification view of the ratio of mature erythrocytes to nucleated cells to determine the degree of bone marrow hyperplasia, which was generally classified into the following five grades: extremely reduced myeloproliferative, reduced myeloproliferative, active myeloproliferative, markedly active myeloproliferative, and extremely active myeloproliferative.

### 2.3 MRI examination

The whole lumbar spine (L1–5) was scanned in the supine position using a Siemens 3.0-Tesla magnetic resonance scanner (Siemens Healthineers, Erlangen, Germany) equipped with a 32-channel spinal matrix coil (22). Spin-echo T1-weighted sequences, T2-weighted sequences, IVIM, and Q-Dixon were performed all in the sagittal plane. IVIM data were obtained during a single spin-echo planar imaging session with 12 distinct b-values. Q-Dixon data were acquired using a Dixon volumetric interpolation breath-holding examination sequence with six echoes, and a seven-peak fat profile model with different lipid components was used in order to better represent the bone marrow variability of fat distribution. Small flip angles were employed to lessen T1 bias, and, following T2* correction, water and fat maps were produced. FF maps were generated automatically. The precise imaging parameters are compiled in **Table 1**.

**Table 1:** Overview of MRI sequence parameters.

	T1W TSE	T2W TSE	Q-Dixon	IVIM
Time to repetition, ms	460	2360	10.30	3000
Time to echo, ms	8.6	98	1.04, 2.50, 3.96, 5.42, 6.88, 8.34	57
b-values, s/mm^2^	–	–	–	0, 10, 20, 30, 50, 70, 100,150, 200, 400, 800, 1000
No. of slices	11	11	12	9
Slice thickness, mm	4	4	3	4
Interslice gap, mm	0.4	0.4	0.3	0.4
Field of view, mm^2^	280 × 280	280 × 280	256 × 256	272 × 272
Acquisition matrix	280 × 280	280 × 280	256 × 240	272 × 170
Voxel size, mm^3^	0.9 × 0.9 × 4.0	0.9 × 0.9 × 4.0	1.3 × 1.3 × 3.0	1.6 × 1.6 × 4.0
Phase-encoding direction	H > > F	H > > F	A > > P	A > > P
No. averages	1	1	3	2–4
Flip angle, degrees	150	160	4	–
Bandwidth, hertz/pixels	240	240	900	1506
Acquisition time, min:s	01:26	01:01	02:14	03:57

*T1W*, T1-weighted; *T2W*, T2 weighted; *IVIM*, intravoxel incoherent motion diffusion; *Q-Dixon*, quantitative Dixon.

### 2.4 MRI analysis

The quantitative characteristics of the Q-Dixon and IVIM images were assessed by two musculoskeletal radiologists with 10 and 15 years of musculoskeletal radiology experience, respectively, who were blinded to the clinical information.

### 2.5 Q-Dixon analysis

Reconstructed FF maps from the Q-Dixon sequence were uploaded to a Siemens imaging workstation (Syngo software version B17; Siemens Healthineers, Erlangen, Germany) in order to extract data using the parameter showing the percentage of fat content in the tissue. On the L2–4 vertebral unit at the center level of the sagittal FF map and four adjacent images on either side, a rectangular area of interest (ROI) was established so as to properly examine the fat component of each vertebral unit. The ROI needs to be as large as possible in order to include all of the cancellous bone while excluding the cortical bone. The final FF value for each vertebral unit was calculated as the average of the five values extracted from the FF map.

### 2.6 IVIM analysis

IVIM raw data were sent to Advantage Workstation (ADW 4.6; GE Healthcare, Chicago, IL, USA) for processing. Using MADC software, background noise was removed, and thresholding and multivalued picture fitting were performed. To produce maps of true apparent diffusion coefficient (D), pseudo-apparent diffusion coefficient (D*), and perfusion fraction (f) values, a bi-exponential model was adopted. As mentioned, the microcirculation-related perfusion fraction is shown using the f parameter. The method used to delineate the ROI followed the Q-Dixon sequence, and the values of D, D*, and f were measured.

### 2.7 Statistical analysis

SPSS 26.0 (IBM Corporation, Armonk, NY, USA) and MedCalc (MedCalc Software Ltd., Ostend, Belgium) were used for statistical analysis. The consistency of parameter measurements between the two observers was analyzed by calculating the interclass correlation coefficient (ICC); ICC values of <0.50 indicated inconsistency, values of 0.50–0.75 indicated moderate agreement, values of 0.75–0.90 indicated good agreement, and values of >0.90 indicated almost complete agreement, respectively. All parameters were tested for Gaussian distribution with the Shapiro–Wilk test. Analysis of variance among the three groups was performed using one-way analysis of variance and *post hoc* comparisons, with *p* < 0.05 considered to indicate statistical significance. The Spearman correlation coefficient was used to analyze the correlation between Q-Dixon–derived and IVIM-derived parameters, the degree of bone marrow hyperplasia. Receiver operating characteristic (ROC) curve analysis was used to analyze statistically significant parameters.

## 3 Results

### 3.1 Basic clinical information and MRI features

**Figure 1:**
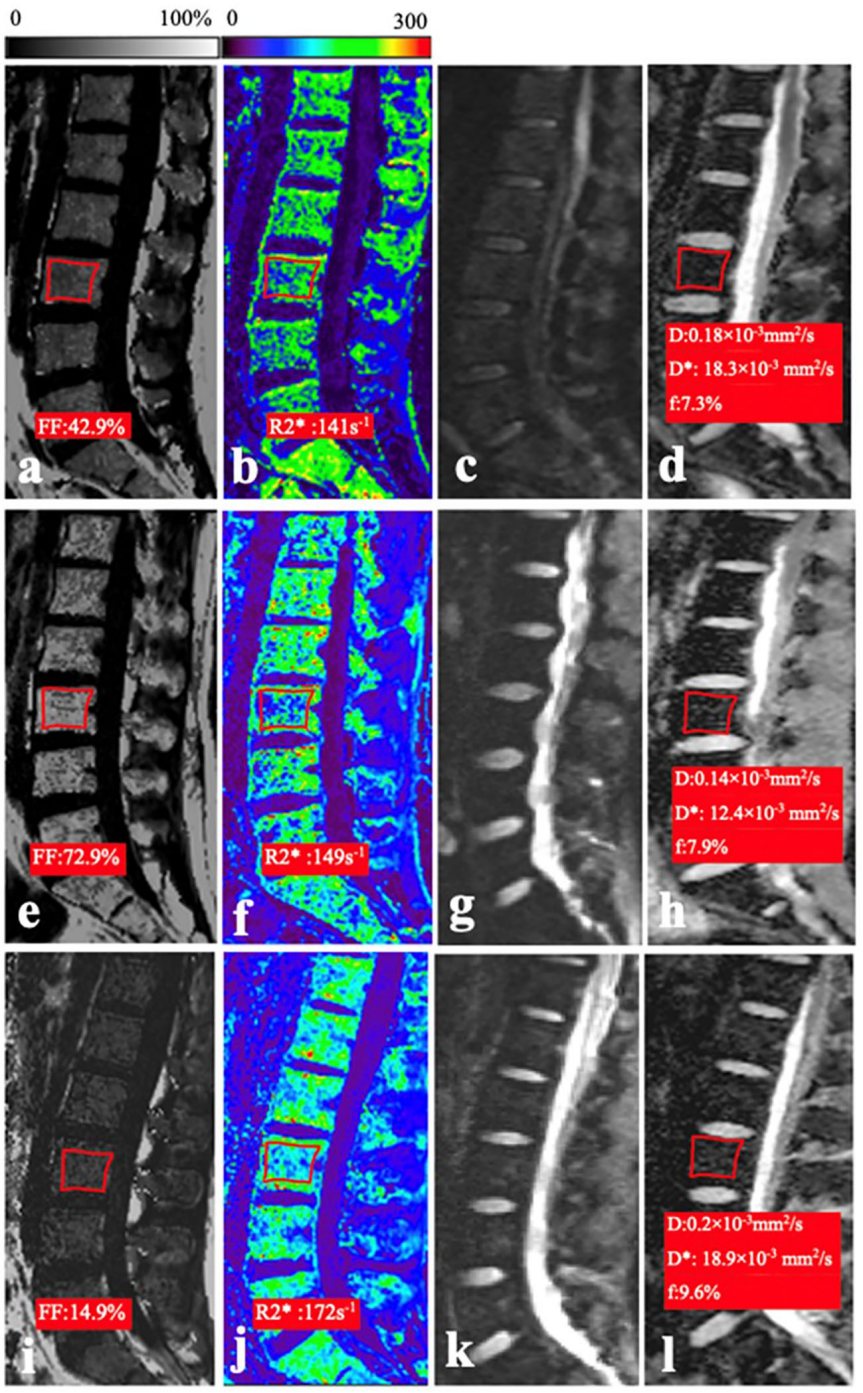
**(A–D)** In a 26-year-old woman of the normal control group, the mean FF, R2*, D, and f values were 42.9%, 141 s^−1^, 0.18 × 10^−3^ mm^2^/s, and 7.3%, respectively. **(E–H)** In a 21-year-old man with AA, the mean FF, R2*, D, and f values were 72.9%, 149 s^−1^, 0.14 × 10^−3^ mm^2^/s, and 14.4%, respectively. **(I–L)** In a 20-year-old man with AML, the mean FF, R2*, D, and f values were 14.9%, 17.2 s^−1^, 0.2 × 10^−3^ mm^2^/s, and 9.6%, respectively.

**Figure 2:**
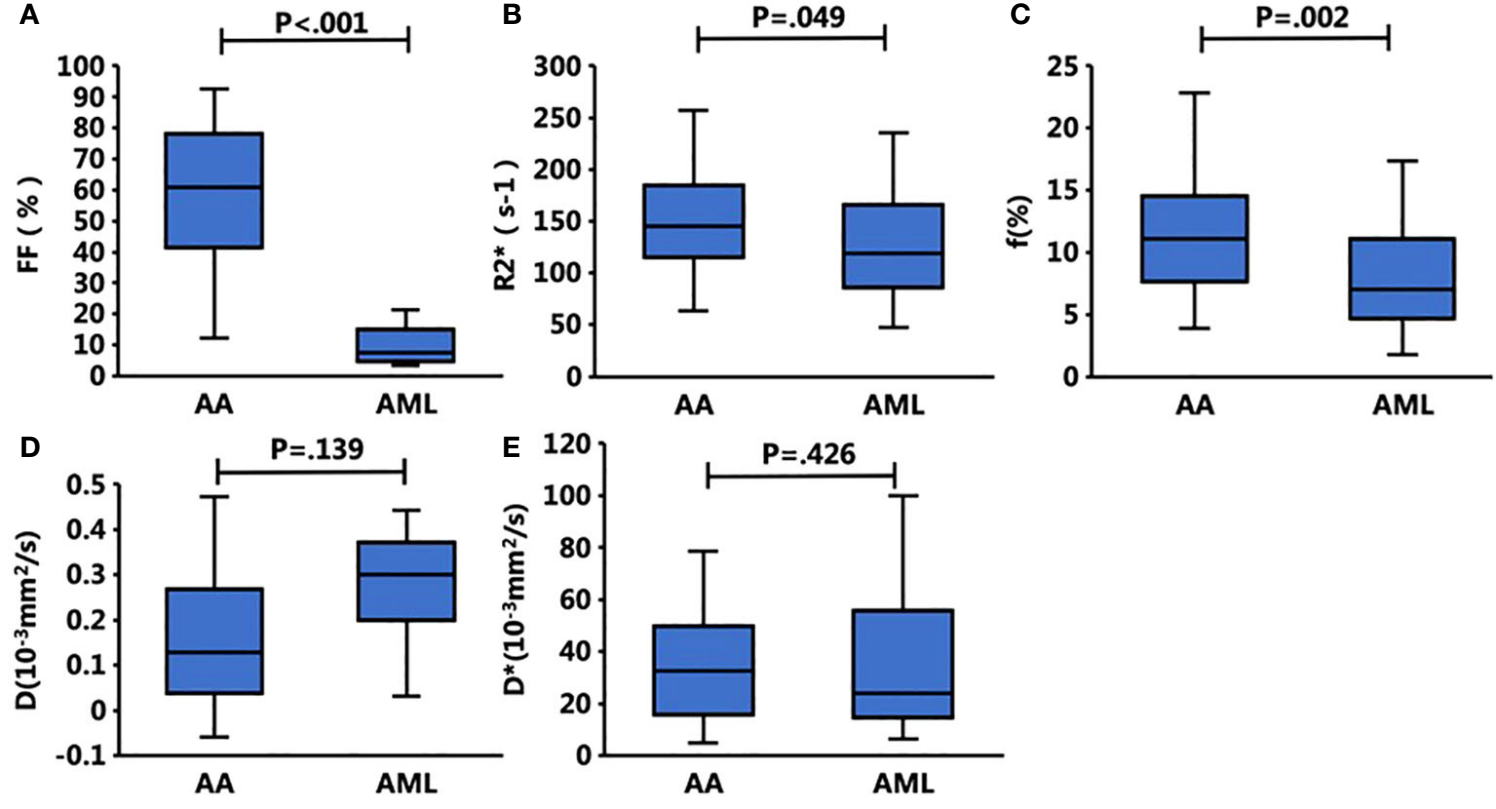
Box plots show the bone marrow Q-Dixon parameters of **(A)** FF and **(B)** R2* and the IVIM parameters of **(C)** f (%), **(D)** D, and **(E)** D* with respect to the presence of AA and AML. The FF, f, and R2* values of the bone marrow were significantly different between these two groups, whereas the other parameters did not differ significantly.

A total of 94 patients were enrolled, including 36 in the AA group (mean age, 35.1 ± 3.1 years), 32 in the AML group (mean age, 53.8 ± 2.7 years), and 26 in the normal control group (mean age, 37.2 ± 3.0 years). Neutrophil counts did not differ significantly between the AA and AML groups (*p* > 0.05). However, there were significant differences between the three groups in terms of age, body mass index, heart rate, hemoglobin level, platelet count, leukocyte count, and reticulocyte count (*p* < 0.05). The age of AA patients was also significantly lower than that of AML patients and control subjects. **Table 2** displays the clinical and MRI features of each group. Meanwhile, the *post hoc* tests of quantitative MRI parameters between the three groups are summarized in **Table 3**. With near-perfect reproducibility, two observers measured the Q-Dixon and IVIM parameters (FF ICC = 0.949, 95% confidence interval [CI] = 0.922–0.967; R2* ICC = 0.947, 95% CI 0.918–0.965; D ICC = 0.995, 95% CI 0.994–0.996; D* ICC = 0.959, 95% CI 0.936–0.973; f ICC = 0.957, 95% CI 0.934–0.972). FF values between the three groups had statistically significant differences (*p* < 0.001); notably, the FF value in the AA group was higher than those in the AML group and control group. The R2* value in the AA group was also higher than that in the AML group (*p* = 0.049), while the R2* value in the AML group was lower than that in the control group (*p* = 0.038). Regarding f values, that of the AA group was higher than that of the AML group (*p* = 0.002). The D and D* values, however, did not vary substantially between the three groups (*p* > 0.05) (**Table 3**; **Figures 1**, **2**).

**Table 2:** Clinical and MRI characteristics of all patients.

	AA (n = 36)	AML (n = 32)	Normal (n = 26)	*p* value
Age (years)	35.1 ± 3.1	53.8 ± 2.7	37.2 ± 3.0	<0.001*
BMI (kg/m^2^)	19.3 ± 0.94	23.94 ± 0.74	24.82 ± 0.78	<0.001*
Heart rate (bpm)	96.96 ± 2.23	98.23 ± 2.95	79.50 ± 3.20	<0.001*
Hemoglobin (g/L)	77.00 ± 3.29	72.19 ± 3.02	137.61 ± 3.18	<0.001*
Leukocytes (10^9^/L)	2.60 ± 0.18	20.02 ± 4.65	6.47 ± 0.35	<0.001*
Platelets (10^9^/L)	54.02 ± 9.12	77.16 ± 14.99	232.44 ± 10.75	<0.001*
Neutrophils (10^9^/L)	2.19 ± 0.94	3.31 ± 0.80	3.82 ± 0.26	0.463
Reticulocytes (10^9^/L)	30.08 ± 5.30	42.59 ± 8.06	2.54 ± 0.35	0.003*
FF (%)	58.90 ± 3.26	13.75 ± 2.67	40.60 ± 1.94	<0.001*
R2* (s^−1^)	156.08 ± 7.31	133.73 ± 10.00	161.21 ± 6.85	0.067
f (%)	11.18 ± 0.62	8.08 ± 0.77	10.14 ± 0.70	0.007*
D (10^−3^ mm^2^/s)	0.16 ± 0.02	3.44 ± 3.16	0.17 ± 0.02	0.283
D* (10^−3^ mm^2^/s)	37.04 ± 3.26	32.38 ± 4.39	42.05 ± 6.14	0.368

BMI, body mass index; D, true apparent diffusion coefficient; D*, pseudo-apparent diffusion coefficient; f, perfusion fraction; FF, fat fraction.

*The result is statistically significant.

**Table 3:** *Post hoc* tests of MRI parameters compared between normal controls and AA and AML patients.

	Normal vs. AA	Normal vs. AML	AA vs. AML
FF (%)	<0.001*	<0.001*	<0.001*
R2* (s^−1^)	0.669	0.038*	0.049*
f (%)	0.311	0.068	0.002*
D (10^−3^ mm^2^/s)	0.996	0.206	0.139
D_*_ (10^−3^ mm^2^/s)	0.424	0.159	0.426

D, true-ADC; D*, pseudo-ADC; f, perfusion fraction; FF, fat fraction.

*The result is statistically significant.

### 3.2 Correlation between the degree of myelodysplasia and MRI parameters

The FF value was negatively correlated with the degree of myelodysplasia (r = −0.597, *p* < 0.001). Also, the f value was negatively correlated with the degree of myelodysplasia (r = −0.454, *p* = 0.004). Finally, the D was positively correlated with the degree of myelodysplasia (r = 0.395, *p* = 0.001).

### 3.3 Correlation between the Q-Dixon and IVIM parameters

The f value was positively correlated with the FF value (r = 0.376, *p* < 0.001). The D value was negatively correlated with the FF value (r = −0.601, *p* < 0.001), whereas the R2* value (r = 0.147, *p* = 0.187) and D* value (r = 0.208, *p* = 0.061) were not significantly correlated with the FF value. Further, the D value was negatively correlated with the R2* value (r = −0.336, *p* = 0.002), while the f value was negatively correlated with the D value (r = −0.320, *p* = 0.003) and positively correlated with the D* value (r = 0.263, *p* = 0.017).

### 3.4 Diagnostic efficacy of the Q-Dixon and IVIM parameters in the prediction of AA

**Figure 3:**
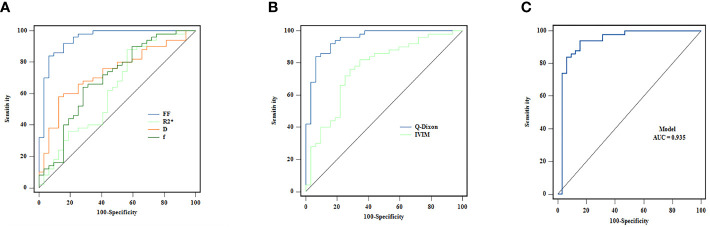
**(A–C)** ROC curves for the individual and combined use of Q-Dixon and IVIM models to discriminate between AA and AML groups.

The single-parameter ROC analysis showed that the FF value had a high area under the ROC curve (0.948) and specificity (84%) in predicting AA. The combined multiparameter model improved the prediction ability of AA, among which the Q-Dixon model was superior to the IVIM model. The two combination models were less effective than the Q-Dixon model in distinguishing AA from AML (**Table 4**; **Figures 3A–C**).

**Table 4:** ROC analysis using different parameters to distinguish between the AA and AML groups.

Parameter	AUC	Cutoff value	Specificity	Sensitivity
**FF**	0.948	34.9%	84.00%	93.75%
R2^*^	0.628	108 s^−1^	43.75%	88%
**D**	0.733	0.14 × 10^−3^ mm^2^/s	87.5%	58%
**f**	0.695	2.88%	71.87%	64%
**Q-Dixon**	0.954	—	85.7%	93.7%
**IVIM**	0.766	—	77.6%	71.9%
**Q-Dixon+ IVIM**	0.935	—	94%	84.37%

AUC, the area under the receiver operating characteristic curve; D, true apparent diffusion coefficient; D*, pseudo-apparent diffusion coefficient; f, perfusion fraction; FF, fat fraction; IVIM, intravoxel incoherent motion diffusion; Q-Dixon, quantitative Dixon.

## 4 Discussion

This research shows that it is possible to discriminate between AA and AML using multiparameter functional MRI. We found that the FF value in the AA group was higher than those in the AML and control groups. The D, D*, and f values of IVIM were associated with the FF and R2* values of Q-Dixon and the degree of bone marrow hyperplasia. Using a ROC curve, we also assessed the diagnostic ability of the two models to discriminate between AA and AML. In a reliable multimodal fat model, the findings showed that Q-Dixon demonstrated clear sensitivity and specificity in the diagnosis of AA.

The main pathological features of AA are decreased hematopoietic tissue, increased adipose tissue, and fewer blood vessels (5, 6); conversely, the primary pathological features of AML are increased abnormal hematopoietic tissue, increased internal microvessels, and decreased adipose tissue (8). The amount of normal hematopoietic tissue is reduced in both AA and AML patients, but the degree of bone marrow proliferation is reduced in AA compared to in AML, while the abnormal proliferation of hematopoietic stem cells in AML leukemia leads to an increase in leukocytes in the peripheral blood. Meanwhile, the values of hemoglobin、platelets in both groups in the present study were higher than those of the normal control group in accordance with the pathological changes in their bone marrow. Moreover, the reticulocyte values, which reflect the degree of bone marrow proliferation, were higher in the AML group than in the AA group.

The T2*-corrected six-echo Q-Dixon approach was used to eliminate the effects of bone trabeculae’s T1 bias and T2*-shortening in order to more precisely estimate the intramedullary FF value using water–lipid separation sequences. We discovered a significant difference between the three groups’ FF values, with that of the AA group being greater than those of the AML and control groups. This finding is in agreement with those of McKinstry (13) and Zeng (16). The results of this study include that the FF value was negatively correlated with the degree of myelodysplasia, reflecting changes in the internal structure of the bone marrow in AA: the lower the degree of bone marrow hyperplasia, the less the amount of internal hematopoietic tissue, and the higher the amount of yellow bone marrow, the higher the FF value, so the FF value can better reflect the change in bone marrow hyperplasia. This study also found that the R2* value was higher in the AA group than in the AML group and it was lower in the AML group than in the control group, respectively, because some of the AA patients required multiple blood transfusions and had more iron deposition in the bone marrow. However, the leukemic patients with AML had a large number of proliferating hematopoietic stem cells and a significant increase in abnormal leukocytes, so their iron required for hematopoiesis was constantly being depleted, which is also in line with the pathological characteristics of the bone marrow in both AA and AML.

As pulse sequences and signal modeling are optimized, the difficulties of diffusion-weighted imaging in bone imaging are diminishing progressively. Diffusion-weighted imaging and IVIM techniques are used to quantify water molecule diffusion and perfusion in bone marrow using MRI. Since traditional mono-exponential ADC values cannot distinguish between true diffusion and perfusion effects in tissues, our study employed a reduced field-of-view technique based on single echo-planar imaging and evaluated the true water molecule diffusion and blood microcirculation in bone marrow using IVIM bi-exponential modeling. Capillary number, flow rate, and vascular permeability influence D and f values. In our study, f values were moderately positively correlated with FF values, whereas D values were negatively correlated with FF values. Due to the large standard deviation of the D* values and the poor repeatability of the signal-to-noise ratio (15, 24), the D* values require further investigation. On the contrary, the f value may become a reliable parameter to reflect bone marrow perfusion non-invasively. In this study, we also found that the f value of the AA group was superior to that of the AML group, suggesting that the perfusion within the bone marrow of the AA group was greater than that of the AML group, which is inconsistent with the results of Fan (15). Sun et al. found clonal hematopoiesis in the bone marrow of 70–80 patients with AA (25, 26), with increased hematopoietic tissue and perfusion within the bone marrow; therefore, bone marrow perfusion may be greater in patients with AA than those with AML, an idea that requires further validation. Our study also found that D values positively correlated with the degree of myelodysplasia and negatively correlated with FF values. The restriction of water molecule dispersion is not significant due to the fact that the greater the degree of bone marrow hyperplasia, the greater the hematopoietic tissue within the bone marrow, the greater the cellular component, and the less the amount of adipocyte component. Some studies have found that the diffusion coefficient of fat (yellow marrow) is 2–3 orders of magnitude lower than that of water (red marrow) (27), therefore, the more adipose tissue, the lower D value, and the higher FF value.

To our knowledge, a limited number of studies have compared the diagnostic efficacy of quantitative fat imaging models and diffusion imaging models. We also investigated the efficacy of Q-Dixon and IVIM models for the diagnosis of AA and found that the Q-Dixon model has high sensitivity and specificity for the diagnosis of AA. Therefore, we suggest that adding the Q-Dixon model to routine spinal MRI protocols for screening patients presenting with peripheral blood reductions may be useful in the future for early diagnosis of AA. The IVIM model has been reported to be valuable in the diagnosis of malignant hematological disorders (6, 15, 21–23). As such, the IVIM model seems promising for identifying both benign and malignant hematological illnesses.

Some limitations should be taken into account when interpreting our results. First, the sample size of this study was small, and future studies need to expand the sample; second, immunohistochemical markers to assess how much vascularity was present were not included in this study to support the diagnostic efficacy of bone marrow perfusion and dispersion; Finally, the spine IVIM still needs to be further optimized, and an optimized spine IVIM would be more useful for exploring microscopic changes in biochemical composition.

In conclusion, the Q-Dixon and IVIM techniques can be used for distinguishing AA from AML, and the FF value can be a reliable predictor of AA diagnosis. Quantitative assessment of bone marrow can be performed without bone marrow aspiration or biopsy by Q-Dixon and IVIM techniques, and can distinguish the degree of proliferation of AA and AML diseases. Q-Dixon can be used to non-invasively evaluate fat content in the bone marrow, and IVIM may reflect different aspects of marrow pathophysiology compared to Q-Dixon. Multimodal MRI is necessary for the visualization of microscopic changes in the bone marrow composition.

## Data Availability

The original contributions presented in the study are included in the article/supplementary material. Further inquiries can be directed to the corresponding authors.
