# Corrigendum: Quantitative Dixon and intravoxel incoherent motion diffusion magnetic resonance imaging parameters in lumbar vertebrae for differentiating aplastic anemia and acute myeloid leukemia

**DOI:** 10.3389/fonc.2024.1472773

**Published:** 2024-10-09

**Authors:** Meidan Hou, Yanan Huang, Jinsong Yan, Guoguang Fan

**Affiliations:** ^1^ Department of Radiology, The First Hospital of China Medical University, Shenyang, China; ^2^ Department of Radiology, The Second Hospital of Dalian Medical University, Dalian, China; ^3^ Department of Hematology, Liaoning Medical Center for Hematopoietic Stem Cell Transplantation, Liaoning Key Laboratory of Hematopoietic Stem Cell Transplantation and Translational Medicine, The Second Hospital of Dalian Medical University, Dalian, China

**Keywords:** acute myeloid leukemia, aplastic anemia, intravoxel incoherent motion, quantitative Dixon, MRI

In the published article, there was an error in affiliation 1. Instead of Department of Radiology, The First Hospital of China Medical University, Dalian, China, it should be Department of Radiology, The First Hospital of China Medical University, Shenyang, China.

In the published article, there was an error in affiliation 2. Instead of Department of Radiology, The Second Hospital of Dalian Medical University, Shenyang, China, it should be Department of Radiology, The Second Hospital of Dalian Medical University, Dalian, China.

The authors apologize for this error and state that this does not change the scientific conclusions of the article in any way. The original article has been updated.

